# An Unusual Case of Refractory Hypoxia on the ICU

**DOI:** 10.1155/2018/3417259

**Published:** 2018-04-17

**Authors:** Caroline Phillips, Clare Harris, Nathaniel Broughton, Thomas Pulimood, Liam Ring

**Affiliations:** ^1^Department of Anaesthesia and Critical Care, West Suffolk NHS Foundation Trust, Bury St Edmunds, UK; ^2^Department of Respiratory Medicine, West Suffolk NHS Foundation Trust, Bury St Edmunds, UK; ^3^Department of Cardiology, West Suffolk NHS Foundation Trust, Bury St Edmunds, UK

## Abstract

We present the case of a 68-year-old gentleman who presented with breathlessness and was found to have NSTEMI, pulmonary oedema, and hypoxia. He remained hypoxic despite appropriate treatment and was found to have preserved LV function and raised cardiac output. CT pulmonary angiogram was negative but a cirrhotic liver was incidentally noted and later confirmed via ultrasound. Bedside examination was positive for orthodeoxia, suggesting a diagnosis of hepatopulmonary syndrome (HPS). The finding of significant intrapulmonary shunting on “bubble” echocardiography confirmed the diagnosis. This patient did not have previously diagnosed liver disease and had largely normal LFTs when the diagnosis was first suspected. We discuss HPS in the context of ICU and suggest how it may be screened for using simple tests. There is no correlation between the presence of HPS and severity of liver disease, yet we believe this is the first reported adult case of HPS on the ICU without previously diagnosed cirrhosis.

## 1. Introduction

Hepatopulmonary syndrome (HPS) is a well-established clinical entity in the context of cirrhotic liver disease and is commonly considered where cirrhotic patients suffer from dyspnoea or hypoxia, also where liver transplantation is being considered. However, it has not previously been reported as a cause of refractory hypoxia in adult patients requiring admission to the Intensive Care Unit (ICU) who do not have a prior diagnosis of liver disease. We describe why HPS might be suspected in hypoxic ICU patients, how it may be screened for using simple bedside assessment, and how a definitive diagnosis can be made.

## 2. Case Report

A 68-year-old gentleman presented to the Emergency Department (ED) with a six-month history of shortness of breath, which had worsened over the previous five days. He had a past medical history of mild Parkinson's disease, insulin-dependent type II diabetes mellitus, hypertension, and angina. He had recently commenced diuretics in the community for possible cardiac failure. He was a minimal drinker and ex-smoker.

Upon arrival in ED, he was in extremis with a respiratory rate of 30, SpO_2_ 0.54 on air, improving to 0.72 with the application of 15 L oxygen via a non-rebreathe mask. His blood pressure and heart rate were normal. An arterial blood gas on oxygen showed pH 7.37, PaO_2_ 5.3 kPa, PaCO_2_ 6.4 kPa, BE −2.7 mEq/L, and lactate 4.4 mmol/L. There were fine bibasal inspiratory crepitations on auscultation of the chest with chest X-ray (CXR) findings consistent with pulmonary oedema. Laboratory investigations showed a significantly raised troponin T of 141 ng/L on admission, later rising to 1118 ng/L (normal range: <28 ng/L). Other than a mildly elevated CRP of 20 mg/L (normal range: <7 mg/L), blood tests were unremarkable including a normal neutrophil count. His initial ECG showed sinus rhythm with mild lateral ST depression but normal T-waves.

**Figure 1:**
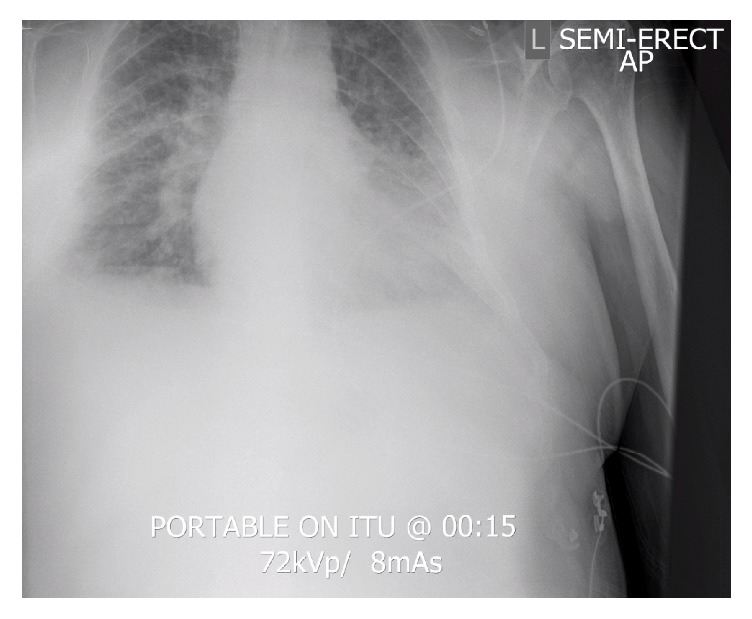
Preintubation CXR shortly after ICU admission.

In addition to anticoagulation with aspirin, clopidogrel, and fondaparinux, the patient received glyceryl trinitrate and furosemide infusions in ED. This led to a good diuresis but persistent hypoxia; PaO_2_ improved only to 6.5 kPa despite CPAP of 7 cmH_2_O. He was, therefore, transferred to the Intensive Care Unit (ICU) for respiratory and circulatory support for a likely non-ST-elevation myocardial infarction (NSTEMI) with hypoxia from presumed cardiogenic pulmonary oedema +/− cardiogenic shock. Lactate had risen to 5.2 mmol/L and base excess deteriorated to −3.2 mEq/L. Noninvasive respiratory support was ineffective and the gentleman required intubation shortly after arrival. A preintubation CXR is shown (Figure 1).

A transthoracic echocardiogram (TTE) showed regional wall motion abnormalities (RWMAs) not present six months earlier, but with normal biventricular size and preserved systolic function. Mild to moderate diastolic dysfunction was found (consistent with chronic hypertension). This suggested the recent myocardial infarction had not resulted in sufficient left ventricular impairment to explain the ongoing hypoxia. Cardiac output monitoring (via LiDCO®) showed a high cardiac output of 8-9 L/min with low vascular resistance; this was also inconsistent with cardiogenic pulmonary oedema and cardiogenic shock.

**Figure 2:**
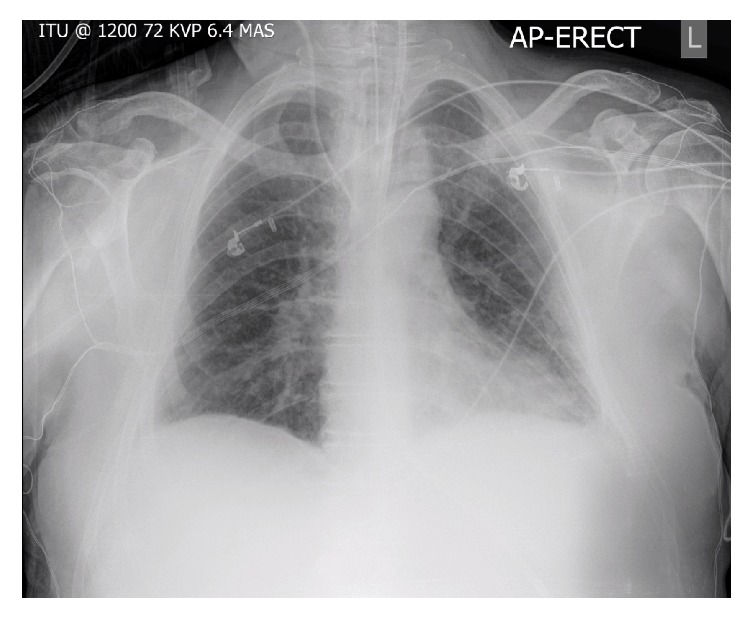
Repeat CXR on ICU day 11; ongoing hypoxia despite successful diuresis.

**Figure 3:**
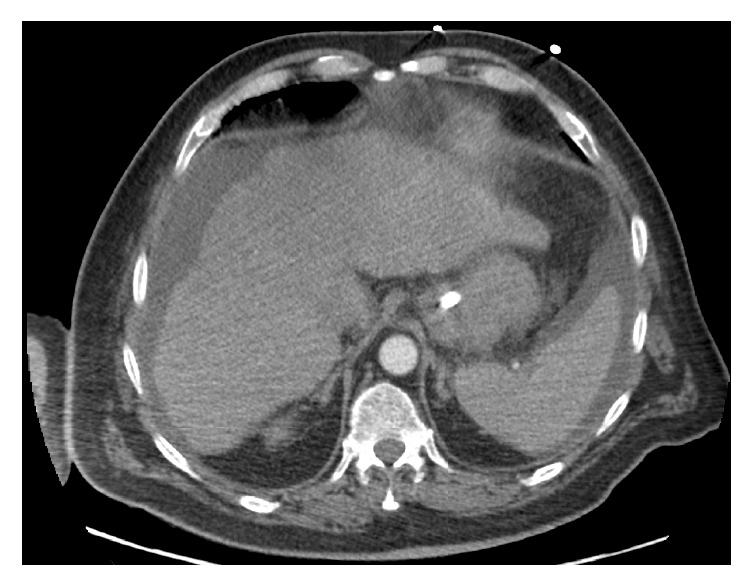
Transverse section of upper abdominal viscera; images acquired during CTPA.

Despite radiological and clinical resolution of pulmonary oedema the oxygen requirements were largely static; one week after admission he was still requiring FiO_2_ 0.6 to maintain PaO_2_ 9-10 kPa; a later CXR is shown (Figure 2). The possibility of a pulmonary embolus was investigated via CT pulmonary angiogram (CTPA). No emboli were visualised in the major pulmonary arteries or their immediate branches; however, poor contrast timing affected imaging of peripheral vessels. The lung parenchyma appeared normal and no pulmonary arteriovenous malformations (AVMs) were seen. Incidental imaging of the upper abdominal viscera showed a nodular and cirrhotic liver with ascites around both the liver and spleen (Figure 3). Subsequent liver ultrasound showed an irregular outline with coarse texture consistent with micronodular cirrhosis. Hepatic vascular flows were normal. Mild finger clubbing was noted during his ICU admission, but no other stigmata of liver disease were present. Of note, this gentleman's liver function tests (LFTs) were largely normal until day seventeen of his admission, except for a stable but mildly raised bilirubin of <35 *μ*mol/L (normal range: 5–17 *μ*mol/L).

**Figure 4:**
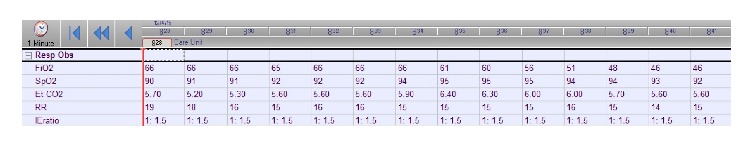
Screenshot of sequential FiO_2_ and SpO_2_ values at one-minute intervals upon moving the patient from a semirecumbent to supine position.

In light of the high cardiac output, persistent hypoxia unexplained by cardiac pathology, and a newly noted cirrhotic liver, the possibility of hepatopulmonary syndrome (HPS) was raised. Marked orthodeoxia was demonstrated; the FiO_2_ required to achieve fixed SpO_2_ rapidly fell from 0.66 to 0.46 upon moving from a semirecumbent to supine posture (Figure 4); however, a test-dose of methylene blue did not improve oxygenation.

**Figure 5:**
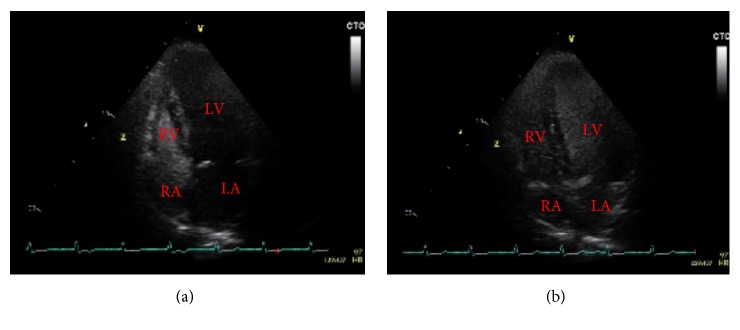
(a) Contrast/“bubble” TTE at time of injection. (b) Contrast/“bubble” TTE showing extensive opacification of the left heart in 4-5 cardiac cycles.

A contrast echocardiogram was performed specifically looking for HPS. This showed extensive opacification of the left heart within four to five cardiac cycles, consistent with an intrapulmonary shunt (Figure 5 & supplementary material (available here)); this confirmed the diagnosis.

The ALT first became elevated at 66 IU/L (normal range: <45 IU/L) on day 17 of the patient's admission, progressively rising to 126 IU/L over the next three days. ALP was 213 IU/L on day 20 (normal range: 40–120 IU/L), having also been normal until three days earlier. His bilirubin remained stable at <35 *μ*mol/L. An ammonia level was measured on day 19 in view of deterioration in LFTs and a failed sedation hold; the level was 205 *μ*mol/L (normal range: 16–60 *μ*mol/L). Admission INR was not recorded as the patient was not warfarinised; also our institution does not routinely record aspartate aminotransferase (AST); we cannot therefore retrospectively calculate a baseline Bonacini score to assess the likelihood of cirrhosis on admission. Platelet count on admission was 196 × 10^9^/L with ALT 17 IU/L at that time. The INR was later measured at 1.5 when liver disease was suspected; however cirrhosis had already been demonstrated via CT and US imaging.

This gentleman's case was discussed with a tertiary hepatology centre—unfortunately there was no medical therapy that could be offered, and he was not a suitable candidate for liver transplantation given his comorbidities. Following discussion with this gentleman's family, the decision was made to withdraw care.

## 3. Discussion

### 3.1. Pseudocardiogenic Hypoxia

This patient was referred to ICU for respiratory and cardiovascular support in the context of an NSTEMI with a large troponin rise and a clinical picture consistent with cardiogenic pulmonary oedema. Given this presentation, left ventricular (LV) impairment and a low cardiac output state might be anticipated. It was therefore surprising to find preserved LV function on echocardiography (despite new RWMAs) alongside a high-output and low-resistance circulation. Whilst the severity of hypoxia at presentation was probably exacerbated by acute pulmonary oedema, the ongoing hypoxia (despite successful diuresis), and consistently hyperdynamic circulation suggested the underlying pathology might be more complicated. Cirrhosis causes systemic vasodilation and therefore reduced systemic vascular resistance [1]. We speculate the LV might have been significantly affected from the presenting NSTEMI and hypoxia; however the abnormally low LV afterload predominated hence the findings of raised output (via LiDCO) and apparent preservation of LV function (via TTE). This may help explain the otherwise contradictory presence of pulmonary oedema (necessarily requiring vascular congestion) despite a background of hyperdynamic pulmonary flow (secondary to HPS). A unifying theory is a “two-hit” process whereby HPS and hypoxia precipitated a type II infarction leading to acute LV impairment and hospital presentation, the hypoxia later reverting to a sole HPS cause once diuresis was successfully achieved. The disproportionate lactate rise at presentation perhaps supports a hypoxic mechanism.

Differential diagnoses leading to high-output cardiac failure were considered (despite no evidence of chronic fluid overload from TTE); none were consistent with this patient's presentation, with the possible exception of Osler-Weber-Rendu syndrome [2]. Visceral AVMs form in this condition, including in the liver (which may lead to portal hypertension and liver failure) and lung (which may lead to shunting and orthopnoea/platypnoea in severe cases). However, this condition was excluded on the grounds that a CTPA demonstrated no pulmonary AVMs and liver Doppler ultrasound showed normal vascular flow and no hepatic AVMs; there were no cutaneous telangiectasia and no history of GI bleeding/iron-deficiency anaemia, nosebleeds, or haemoptysis. This gentleman did not have a history of alcohol excess or other risk factors for thiamine deficiency which would have led us to consider wet beriberi.

### 3.2. Hepatopulmonary Syndrome

Hepatopulmonary syndrome is defined by the triad of liver failure, abnormal arterial oxygenation, and intrapulmonary vascular dilatations [3]. The diagnosis is usually made in the context of hypoxaemia in known cirrhosis, when other cardiopulmonary causes have been excluded and pulmonary vasodilatation is demonstrable [4].

HPS has two subtypes; type I is more common and results from diffuse pulmonary vascular dilatation with the normal capillary diameter of 7–15 *μ*m commonly increasing up to 10-fold, and up to 500 *μ*m on occasion [4, 5]. This gross dilatation markedly accelerates pulmonary transit time and impairs the oxygenation of pulmonary capillary blood. This results in functional shunting; this shunt is unique because it partially responds to supplemental oxygen. Type II HPS results from discrete arteriovenous communications (visible on CTPA); this is true shunt which does not respond to supplemental oxygen [4]. Our patient had type I HPS and we speculate that the poor CTPA contrast timing may have resulted from his abnormal pulmonary haemodynamics.

The precise mechanism underlying type I HPS is uncertain, but vasoactive agents increased in liver disease that dilate the pulmonary circulation (either directly or indirectly) have been identified; these include endotoxin, nitric oxide, and TNF-*α*. Some limited improvement in oxygenation has been demonstrated following treatment with antibiotics, methylene blue, and pentoxifylline, respectively [6–8]. The observation that pathological vasodilatation of the pulmonary bed may be attenuated without improved oxygenation however suggests a more complex pathophysiology, and recently, impaired hypoxic pulmonary vasoconstriction has been implicated [9].

The diagnosis of HPS does not have to be made in the context of overt or previously diagnosed liver disease. There is, in fact, no relationship between the presence or severity of HPS and the severity of liver disease [3]. In Abrams et al.'s comparison of the diagnostic utility of contrast echocardiography and lung perfusion scan in patients with HPS, they found a similar prevalence of positive contrast echocardiograms (i.e., with intrapulmonary vascular dilatations present) across a range of aetiologies and severities [10]. Similarly Krowka et al. noted that* “severe HPS can occur in patients with mild liver disease”* [11].

Despite this, we are only aware of one adult case having been previously published regarding a patient presenting with hepatopulmonary syndrome without known liver failure (although there are numerous paediatric cases); this recent adult case was not on the ICU [12–16]. Therefore, our patient's case uniquely demonstrates the importance of considering the diagnosis of hepatopulmonary syndrome in cases of unexplained hypoxia in adults on the ICU.

### 3.3. Orthodeoxia in ICU

Whilst dyspnoea is a typical presenting feature of HPS, other mechanisms for respiratory deterioration exist in patients with liver disease, including anaemia and ascites. Signs of chronic hepatic failure, such as digital clubbing and spider naevi are common in HPS, but nonspecific. The presence of platypnoea-orthodeoxia (in the context of liver disease) is highly specific for HPS [17].

Platypnoea-orthodeoxia is an unusual phenomenon characterised by positional dyspnoea (platypnoea) and hypoxaemia (orthodeoxia) exacerbated in the sitting position/improved in the supine position. Orthodeoxia is defined as a PaO_2_ decrease of ≥5%, or ≥4 mmHg (≥0.53 kPa) when changing position from supine to upright [18]. Orthodeoxia can most easily be demonstrated in patients on ICU by improved oxygenation upon on laying the patient flat, that is, by reducing FiO_2_ whilst achieving constant SpO_2_/PaO_2_.

There are several causes of platypnoea-orthodeoxia in the general population that can be divided into cardiac, respiratory, abdominal, and autonomic groups. The differential narrows for intubated and ventilated patients to either intracardiac or intrapulmonary shunts. Intracardiac shunting is more common; however, in the context of chronic liver disease, orthodeoxia is highly specific for HPS [17]. Both intracardiac and intrapulmonary shunts can be identified on contrast echocardiography, making it a good discriminator for the aetiology of platypnoea-orthodeoxia in ICU patients.

HPS preferentially affects basal lung vasculature. Moving from a semirecumbent to supine position will necessarily reduce perfusion to lung bases via gravitational effect. This increases pulmonary circulation to less-affected vascular beds thereby reducing shunt fraction and improving oxygenation; this mechanism is thought to underlie platypnoea-orthodeoxia in the context of HPS [19].

### 3.4. Role of Contrast Echocardiography

Contrast echocardiography is the use of injected agitated saline to produce bubbles of >15 *μ*m in diameter and then to observe for the presence of microbubbles in the left heart [4]. It can be used to identify both intrapulmonary and intracardiac shunts. An intrapulmonary shunt is defined by microbubbles seen in the left heart after the third beat (and usually before the sixth), due to passage of microbubbles through an abnormally dilated vascular bed. Conversely, visualisation of microbubbles in the left atrium in fewer than three beats is indicative of an intracardiac shunt; no visualisation is consistent with no shunt, as the microbubbles are trapped and absorbed during the first pass through normal pulmonary vasculature [20].

Abrams et al. showed contrast echocardiography to be superior to lung perfusion studies to screen for intrapulmonary vasodilatation in patients with cirrhosis, and it has since been recommended by the European Respiratory Society Task Force of Pulmonary-Hepatic vascular disorders as the first line screening modality for HPS [10, 21]. Abrams et al. noted intrapulmonary vascular dilatation in up to 30% of patients with cirrhosis, although only in 10% was this severe enough to cause hepatopulmonary syndrome [10].

## 4. Conclusion

Although patients with HPS typically present with hypoxia on the background of chronic liver disease, a presentation with hypoxia (and the complications of hypoxia, as in this case) may be their first. As there is no correlation between the severity of liver failure and the presence of HPS, we advocate the inclusion of HPS into the differential diagnosis for all patients with unexplained hypoxia on ICU. This differential diagnosis should prompt bedside assessment for orthodeoxia; HPS is excluded where orthodeoxia is not found. Patients with unexplained hypoxia will routinely have TTE performed; we advocate this investigation to be extended to contrast echocardiography for patients in whom orthodeoxia has been demonstrated. Should the contrast TTE demonstrate intrapulmonary shunting, a full assessment of liver function should be made.

The first suggestion of liver disease in this case came from the incidental inclusion (and reporting) of the abnormal liver via CTPA. Since a common indication for CTPA in ICU patients is unexplained hypoxia, we speculate whether the imaged field should be* deliberately* extended to the upper abdomen for all CTPA investigations in this cohort. We certainly advocate that where the liver is imaged (either intentionally or not) the appearance should be reported. Abnormal liver appearance in the context of hypoxia should be a further prompt for the bedside assessment of orthodeoxia (and possible consequent contrast TTE).

Any patient in whom HPS is suspected should be discussed with a tertiary centre regarding further assessment and suitability for liver transplantation.

## Supplementary Materials

Supplementary Materials Transthoracic contrast “bubble” echocardiogram for this patient.
